# The Protective Effects of MSC-Derived Exosomes Against Chemotherapy-Induced Parotid Gland Cytotoxicity

**DOI:** 10.1155/ijod/5517092

**Published:** 2025-04-03

**Authors:** Mahmoud M. Bakr, Mahmoud Al Ankily, Mohamed Shamel

**Affiliations:** ^1^General Dental Practice, School of Medicine and Dentistry, Griffith University, Gold Coast, Queensland 4215, Australia; ^2^Faculty of Dentistry, Oral Biology Department, The British University in Egypt, Cairo, Egypt

**Keywords:** chemotherapy, exosomes, fluorouracil, parotid gland, stem cells

## Abstract

**Background:** Fluorouracil (5-FU) is one of the most popular chemotherapeutic agents used in various cancer therapy protocols. Cell-free therapy utilizing exosomes is gaining increased popularity as a safer option due to concerns over potential tumor progression following stem cell therapy.

**Methods:** Parotid glands of albino were treated with a single bone marrow mesenchymal stem cell (BMMSC)-derived exosomes injection (100 μg/kg/dose suspended in 0.2 mL phosphate-buffered saline [PBS]), a single 5-Fu injection (20 mg/kg), and BMMSC-derived exosomes plus 5-FU and compared to control group (daily saline injections). After 30 days, the parotid glands were examined using qualitative histological evaluation, immunohistochemical evaluation using rabbit polyclonal mouse antibody to Ki-67, caspase 3, and *iNOS*, as well as quantitative real-time polymerase chain reaction (RT-PCR) to evaluate gene expression of *TGFβ1*, *TNF-α*, and *BCL-2*.

**Results:** Histological examination of the parotid gland revealed that BMMSC-derived exosomes restored the glands' architecture and repaired most of the distortion created by 5-FU. Immunohistochemical expression of tumor proliferation and cell death markers were restored to normal levels in the exosome-treated groups that were similar to the control group. Furthermore, BMMSC-derived exosomes reversed the effects of 5-FU on quantitative gene expression levels and showed a significant decrease in *TNF-α* (*p* < 0.001) and a significant increase in *TGFβ* (*p* < 0.0001) and *BCL-2* (*p* < 0.05) when compared to 5-FU treatment.

**Conclusion:** Within the limitations of the current study, BMMSC-derived exosomes have the potential to counteract the cytotoxic effects of 5-FU on the parotid glands of rats in vivo. Further studies are deemed necessary to simulate clinical scenarios.

## 1. Introduction

Fluorouracil (5-FU) is a potent chemotherapy drug that treats a diverse range of cancerous conditions [1]. The FDA has approved the use of 5-FU for the treatment of gastric adenocarcinoma, pancreatic adenocarcinoma, breast carcinoma, and colorectal adenocarcinoma. Moreover, 5-FU is licensed by the FDA for topical application in treating various cancers, dermatologic diseases, and superficial basal cell carcinomas when alternative treatment options are not feasible [2]. 5-FU is an inhibitor of thymidylate synthase (TYMS), leading to a shortage of thymidine monophosphate (dTMP), one of the three nucleotides that make up thymine [3]. This mechanism also contributes to the formation of oxidative stress (OS), resulting in decreased DNA replication, damage to the genome, and apoptosis [4]. Earlier studies indicate that 5-FU can alter saliva's pH and ionic composition and diminish salivary enzyme levels and the salivary flow rate, leading to xerostomia in humans [5]. Additionally, it was shown that 5-FU causes OS in the glandular structure of submandibular and sublingual glands [6].

Regenerative medicine is a rapidly advancing area within the contemporary medical sciences [7]. Stem cell-based therapy, a form of regenerative medicine, has garnered significant interest due to its potential to provide alternative treatments for patients with previously untreatable conditions [8, 9]. Since then, numerous clinical trials have been registered, encompassing a broad range of medical issues, including musculoskeletal and neurological disorders, immunological diseases, hematological dysfunctions, and degenerative ailments [10, 11]. Nevertheless, specific experiments have proven unsuccessful in demonstrating advantageous outcomes in the clinical setting. This can be attributed to the inherent constraints of stem cell therapy, including infusion toxicity, immunogenicity, tumorigenic capabilities, and ethical concerns [12]. Exosomes, released by nearly all types of cells, including stem cells, have been proposed as a safer and more adaptable substitute for stem cell therapy [13].

Like all other cells in the human body, stem cells secrete exosomes to facilitate communication, acting as intercellular messengers. Exosomes are small vesicles enclosed by a liposomal membrane, measuring approximately 30–200 nm in diameter [14]. Stem cell-derived exosomes (SC-Exo) have comparable therapeutic properties to the cells they originate from [15]. Unlike stem cells, exosomes cannot undergo self-replication, alleviating worries over tumor development following stem cell transplantation. Therefore, SC-Exo serve as a powerful alternative to stem cell therapy while avoiding the drawbacks of actual stem cells [16]. The advantageous impacts of exosomes produced from mesenchymal stem cells (MSCs-Exo) on tissue regeneration have garnered considerable attention for their utilization in cell-free therapies. MSC-Exo has been demonstrated to have beneficial functions in restoring and regenerating many tissues, such as cartilage, skin, and skeleton [17, 18].

Bone marrow-derived MSCs are highly promising for clinical applications due to their superior flexibility and regenerative capabilities in laboratory settings. This study aimed to harness the regenerative potential of exosomes derived from bone marrow mesenchymal stem cells (BMMSCs) to alleviate the detrimental cytotoxic effects of 5-FU on the parotid salivary glands. This study holds significant significance as it centers on the comparatively underexplored domain of salivary gland regeneration, specifically in the context of damage caused by chemotherapy.

## 2. Methods

### 2.1. Study Setting and Sample Selection

This study was approved by the ethical committee at the British University in Egypt (EX-2401). Thirty-two male albino rats weighing 150–200 g were used in this study according to sample size calculation using power analysis. Total sample size *N*=(1.96)^2^ × (5.65)^2^/(2)^2^=31.903 ≈ 32 samples. Rats were kept in a 12 h/12 h dark and light cycle with free food and water adlibitum access.

#### 2.1.1. Exosomes Preparation

A—Isolation and Culture of BMMSCs: Bone marrow cells were obtained from the tibia of albino male white rats. Phosphate-buffered saline (PBS) was used to wash the cells. The cells flushed were put on 15 mL of Ficoll-Paque (Gibco Invitrogen, Grand Island, NY) and centrifuged at 400 × *g* for 35 min. Cells at the interface were aspirated and washed twice in sterile PBS and centrifuged for 10 min at 200 × *g* 5 °C. The isolated BMMSCs were cultured in a DMEM medium supplemented with 10% fetal bovine serum (FBS) and penicillin. Cells of the third passage (P3) were used in the study.

B—Identification of BMMSCs: Morphology of BMMSCs under an inverted microscope and flow cytometric analysis for the positivity of cluster of differentiation (CD) 73, 90, and 105 and negativity of CD 34 and 45 were assessed [19].

C—To isolate exosomes, BMMSCs were cultured in exosome-depleted FBS (prepared by ultracentrifugation at 100,000 × *g* for 18 h) for 48 h before collecting the conditioned media. This step was taken to minimize contamination from FBS-derived extracellular vesicles (EVs).

The exosomes were isolated from the supernatants using a standard ultracentrifugation protocol. First, the samples were centrifuged at 2000 × *g* for 30 min to remove cells and debris, followed by 10,000 × *g* for 30 min to eliminate subcellular components. Next, exosomes were pelleted by ultracentrifugation at 100,000 × *g* for 70 min. The obtained exosome pellet was resuspended in 0.01 M PBS and subjected to an additional purification step by ultracentrifugation at 100,000 × *g* for 70 min. Finally, the purified exosomes were stored at −80 °C for future use [20].

Exosomes were identified using transmission electron microscopy (TEM) for morphological analysis, confirming the presence of vesicles within the characteristic exosomal size range of 30–150 nm. Additionally, flow cytometry was performed to assess CD63 and CD81 positivity, validating the exosomal nature of the isolated vesicles [21, 22].

### 2.2. Animals Grouping and Treatment Protocol

The animals were grouped randomly into four main groups: a control group and three experimental groups, eight rats each. The groups were divided and treated as follows:

1. Group I (negative control group): eight rats receiving saline daily via oral gavage for 30 days. This group served as a physiological control that closely mimicked the natural route of exposure for systemic effects, given that chemotherapy-induced toxicity can affect both local and systemic pathways.
2. Group II (exosomes group): containing eight rats receiving a single injection of exosomes (100 μg/kg/dose suspended in 0.2 mL PBS). This group served as positive control [23].
3. Group III (5-FU group): Containing eight rats, each receiving a single IP injection of 5-FU drug (20 mg/kg) [24].
4. Group IV (5-FU + exosomes group): containing eight rats, the rats were treated as group III, then on day 2, they received a single injection of exosomes (100 μg/kg/dose suspended in 0.2 mL PBS) through the tail vein [23].

After the experiment period, the animals were sacrificed with an extra dose of anesthesia, and the parotid glands from each rat were excised. The tissues were prepared for histological, immunohistochemical, and polymerase chain reaction (PCR) evaluations.

### 2.3. Methods of Evaluation

#### 2.3.1. Histological Evaluation

After the fixation of the gland specimens in 10% neutral buffered formalin, the specimens were dehydrated by their immersion in successive ascending concentrations of ethanol (60%, 70%, and 95%). Then, they will be infiltrated with molten paraffin wax (55%) to be embedded later in paraffin wax blocks. The blocks will be cut with a microtome to obtain 4–5 µm thickness sections mounted to glass slides stained with hematoxylin and eosin. After staining, the slides will be examined under a light microscope.

#### 2.3.2. Immunohistochemical Evaluation

Other sections of 5 microns thickness were processed and stained using rabbit polyclonal mouse antibody to Ki-67, caspase 3, and iNOS. Slides were examined with ZEISS primo star light microscopy photographed by Tucsen IS 1000 10.0 MP camera. The mean area percentage expression of the markers was measured using ImageJ software.

#### 2.3.3. Quantitative real-time PCR (RT-PCR)

RNA isolation was performed using a Total RNA extraction kit according to the manufacturer's protocol. Then, total RNA will be reverse transcribed using a cDNA synthesis kit. Finally, SYBER Green qPCR master mixes were used in QIAGEN`s RT-PCR cycler to evaluate gene expression of *TGFβ1*, *TNF-α*, *and BCL-2*. The genes were normalized to *β*-actin. Primer sequences are shown in Table 1.

#### 2.3.4. Statistical Analysis

The statistical analyses were conducted using GraphPad Prism software version 6.0. The results are reported as the mean value and the standard deviation (SD). The statistical analysis was performed using one-way ANOVA, followed by a post-hoc Tukey's HSD test. A significance level of *p*-value  < 0.05 was employed to determine the significance of all results. The experiments were replicated in triplicates, each with three to six repetitions.

## 3. Results

### 3.1. Isolation, Identification, and Characterization of BMMSCs

**Figure 1:**
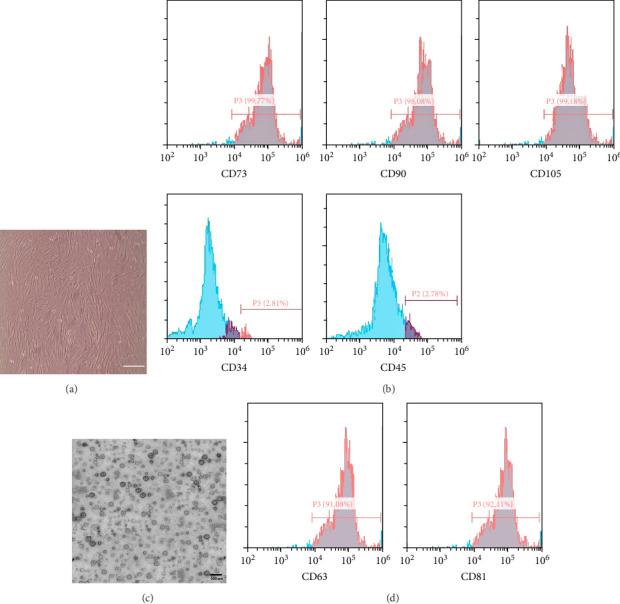
Characterization of BMMSCs by inverted microscope showing spindle-shaped and confluent cells (A) and flowcytometry analysis showing positive expression of CD73, 90, and 105 and negative expression of CD34 and 45 (B). Characterization of exosomes by transmission electron microscope (C) and flow cytometry analysis showing positive expression of CD63 and 81 (D).

BMMSCs were isolated and identified by their spindle-fusiform shape and formation of colonies (Figure 1A). Moreover, characterization by flow cytometry analysis revealed that BMMSCs have a positive expression for CD73, CD90, and CD105 surface markers and a negative expression for CD34 and CD45 (Figure 1B).

### 3.2. Characterization of Exosomes

The identification of exosomes derived from BMMSCs was achieved using TEM. These exosomes were characterized by their consistent size range of 30–100 nm and spherical shape (Figure 1C). Furthermore, the positive expression of specific markers CD63 and CD81 was confirmed using flow cytometry analysis (Figure 1D).

### 3.3. Histological Results

**Figure 2:**
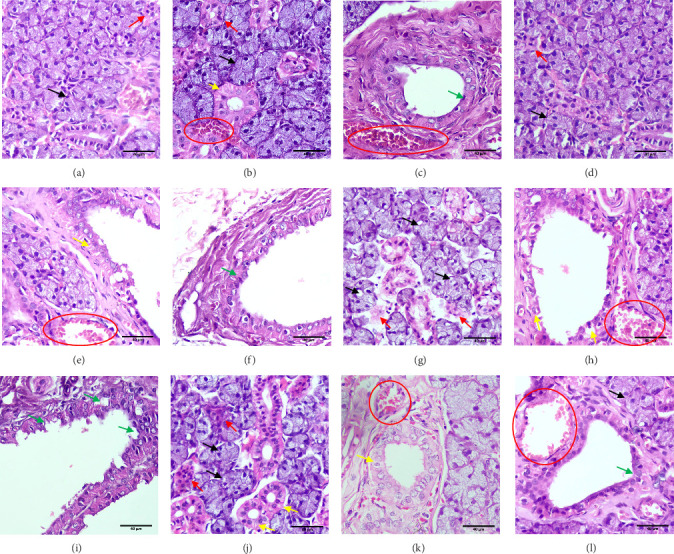
Histological analysis H&E stain showing Group I (A–C), Group II (D–F), Group III (G–I), Group IV (J–L), consisting of pure serous acini (black arrows), intercalated ducts (red arrows), striated ducts (yellow arrows), excretory duct (green arrows), and blood vessels (red circles). Original magnification 400x.

Analysis of H&E-stained sections of parotid glands from the negative control group demonstrated a consistent and typical histological composition. The glandular parenchyma exhibited the characteristic arrangement of lobular structures, consisting of pure serous acini and ducts. The acini had a central lumen surrounded by pyramidal-shaped cells characterized by rounded vesicular nuclei. The striated ducts were lined by columnar epithelium with basal striations (Figure 2). The histological evaluation of Group II revealed mostly typical histological characteristics of the parotid gland. The glands were partitioned into lobes and lobules by connective tissue septa. The acini were of average size and had pyramidal cells with moderately basophilic cytoplasm and darkly stained basophilic rounded basal nuclei. The acinar cells surrounded a clearly defined constricted lumen. A minimal number of deteriorated acini were observed. There were prominent blood veins and a notable scarcity of leaked red blood cells (Figure 2). The histological analysis of Group III revealed a significant reduction in the size of acini and a substantial decrease in their quantity. Several acini exhibited degeneration. Most acini had deformed morphology, characterized by poorly defined cellular boundaries, cytoplasmic vacuolations, decreased cytoplasmic basophilia, and loss of eosinophilia at the apices of the cells. The presence of condensed, variable-in-shape, intensely stained, and fragmented nuclei was seen in most cells. The striated duct cells clearly saw a reduction in both their height and the presence of basal striations, along with the development of cytoplasmic vacuolations. Several of their nuclei exhibited karyolysis, while others had pyknosis and hyperchromatism. The pseudo-stratification of the epithelial lining of the excretory ducts has been distorted (Figure 2). The glandular components of the group treated with exosomes exhibited a typical acinar size and structure, as depicted in (Figure 2). The cells lining the intralobar ducts were observed to be appropriately structured. Most striated duct cells exhibited a columnar shape with centrally located nuclei. These cells had eosinophilic basal striations and acidophilic cytoplasm. Several dilated and congested blood vessels were observed. Reduced fibrosis was noted around the excretory duct (Figure 2).

### 3.4. Immunohistochemistry

**Figure 3:**
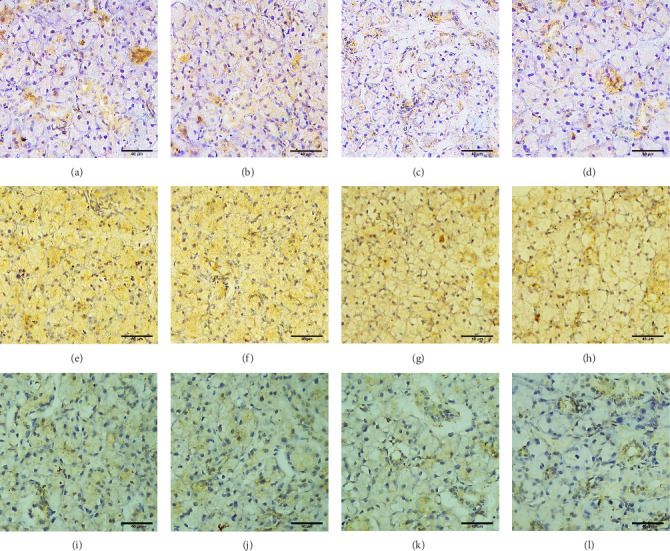
Immuno-stained sections of iNOS showing Group I (A), Group II (B), Group III (C), Group IV (D), Ki-67 showing Group I (E), Group II (F), Group III (G), Group IV (H), and caspase 3 showing Group I (I), Group II (J), Group III (K), Group IV (L). Original magnification 400×.

*iNOS*, Ki-67, and caspase 3 immuno-stained sections of the parotid of the control and positive control rats showed weak positive immunoreaction in the cytoplasm of a few duct cells and cells surrounding the acini (Figure 3A,B,E,F,I, J). The 5-FU group revealed strong positive immunoreaction to *iNOS*, Ki-67, and caspase 3 in the cytoplasm and nuclei of nearly all duct epithelial cells and cells surrounding the acini (Figure 3C,G,K). The increased immunoreaction of the 5-FU group to *iNOS*, Ki-67, and caspase 3 antibodies was statistically significant compared to the control groups. In the exosome-treated group, *iNOS*, Ki-67, and caspase 3 immunoreaction decreased compared to 5-FU (Figure 3 D,H,L); this difference was significant (*p*  < 0.05). A nonsignificant (*p*  > 0.05) difference was found comparing exosomes treated group to the control.

### 3.5. RT-PCR (q RT-PCR)

**Figure 4:**
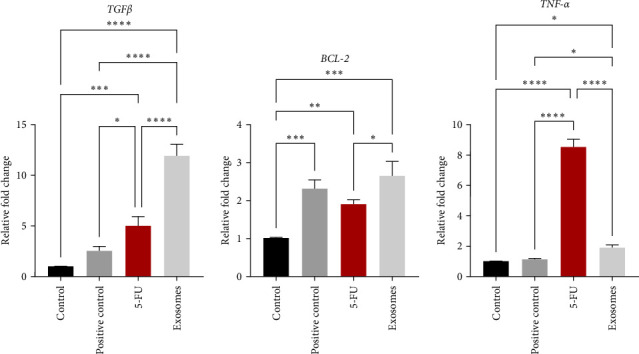
RT-PCR analysis of *TGFβ*, *BCL-2 and TNF-α* genes in the experimental groups. Levels of significance were *⁣*^*∗*^*p* < 0.05, *⁣*^*∗∗*^*p* < 0.01, *⁣*^*∗∗∗*^*p* < 0.001, and *⁣*^*∗∗∗∗*^*p* < 0.0001.

**Figure 5:**
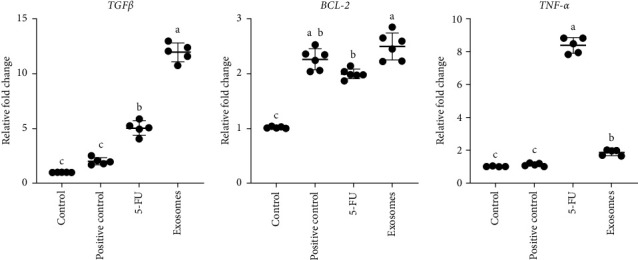
Scatter plot showing the correlation and variability between each sample of the RT-PCR within the individual groups. The groups that have been marked by different lowercase letters (a, b or c) showed significant differences at *p* < 0.05 based on Tukey HSD test.

*T*-test analysis demonstrated statistically significant increases in the quantitative gene expression levels of *TGFβ and TNF-α* in the 5-FU treated group compared to positive and negative control groups. Furthermore, there was a statistically significant increase in the quantitative gene expression levels of *BCL-2* in the 5-FU treated group compared to the negative control group. No significant differences were observed in *BCL-2* expression between 5-FU and positive control groups. On the other hand, there was a significant increase (*p*  < 0.05) in the expression of *TGFβ* and a significant decrease of *TNF-α* (*p*  < 0.0001) in the (5-FU + exosomes) treated rats compared to the 5-FU group (Figure 4). *BCL-2* gene expression was significantly (*p*  < 0.05) elevated in the (5FU + exosomes) treated group compared to the 5-FU group. All gene expression levels were significantly higher in the (5-FU + exosomes) groups when compared to the positive and negative controls, with the exception of the *TNF-α* expression in the positive control group that showed similar values to the (5-FU + exosomes) group (Figures 4 and 5).

## 4. Discussion

Exosomes are a subcategory of EVs; as a result, both share similar properties and besides therapeutic applications, regulate bodily functions within the immune system and nervous system, and are used for diagnosis and detection of cancer progression [25]. Specific to the oral cavity, oral MSC-derived EVs play a significant role in periodontal bone remodeling [26, 27] and exosomes derived from the apical papilla promote dentino–pulp complex regeneration [28]. It is also possible to isolate EVs including exosomes from dental pulp stem cells (DPSCs) of extracted periodontally affected teeth [29]. Furthermore, vesicular secretory products from dental and gingival MSCs promote neural and nerve regeneration [30, 31]. It has been reported that EVs derived from MSCs isolated from tissues within the oral cavity have more superior properties than those isolated from MSCs elsewhere in the body, besides the advantage of ease of collection through a less invasive procedure [32]. A study investigated DPSC-derived exosomes on the restoration of salivary gland functions in Sjogren's syndrome showed promising results [33]. Based on the above, we hypothesize that the negative effects of 5-FU could have been reversed or inhibited more significantly if we used MSCs that were derived from the oral cavity or teeth rather than BMMSCs isolated from the rat's tibia. This hypothesis is backed up by observations related to reduction of the dental pulp inflammation which was best achieved with exosomes derived from DPSCs rather than BMMSCs due to the difference in the immunomodulatory effects between both types of exosomes [34]. We also acknowledge that FBS contains a significant number of EVs, which can introduce exogenous RNA, proteins, and signaling molecules that may interfere with the characterization and biological effects of BMMSC-derived exosomes. To minimize FBS-derived EV contamination, we used exosome-depleted FBS (prepared by ultracentrifugation at 100,000 × *g* for 18 h) in all culture conditions prior to exosome isolation. This approach is widely recommended in the field to remove the majority of bovine EVs that could otherwise co-purify with BMMSC exosomes.

The current study investigated the potential effects of BMMSC-derived exosomes on counteracting the cytotoxic effects of 5-FU on the parotid salivary glands. A recent study discussed the vast application for the use of exosomes in targeted oral cancer therapy as well as being a biomarker for early diagnosis of oral diseases [35]. Exosomes control tumors' microenvironment and play a significant role in regulating its progression and growth [36]. Therefore, utilizing exosomes could be a powerful tool in manipulating tumors' microenvironment and could be useful in creating unfavorable conditions for cancer progression. The results from the current study could be expanded in the future to assist with management of salivary gland neoplasms through altering and/or augmenting salivary gland exosomes [37]. Similarly, the term “Engineered Stem Cells Exosomes” has been introduced in the field of oral and maxillofacial wound healing as a promising adjunctive treatment protocol [38]. These innovative measures have the potential, however, are still experimental. The absence of clinical trials remains a challenge slowing down the advancement in this field [39, 40].

The mechanism of action of exosomes is multimodal and is mainly correlated to the contents that exosomes carry as cargo along its journey from the host to the target tissue [41]. It has been proven that 98% of miRNAs of the stem cells are carried by exosomes and microvesicles (MVs) that are derived from those cells [42]. This indicates that the exosomes will carry most if not all the inherent properties of their stem cells of origin. One of the possible mechanisms for exosomes' action is immunoregulation. In studies related to suppression of cytokine storms in the management of COVID-19, it was found that MSCs-Exo stimulated the release of *IL-4*, *IL-10*, and *TGF-β* [43]. Another study on labial gland-derived MSCs and their exosomes promoted the secretion of *TGF-β* by T-cells [44]. Finally, human DPSCs regenerated salivary gland defects in diabetic rats through increasing *TGF-β* serum levels [45]. Those studies were in alignment with the present study, where *TGF-β* expression increased with BMMSC-derived exosomes. However, the question raised is whether the use of native MSCs from salivary gland tissues in our experiment would have made a significant further improvement in the results. Another possible mechanism of action of MSC-derived exosomes is related to their ability to regulate the activity of fibroblasts, keratinocytes, and endothelial cells [46]. In addition to the above, angiogenesis was linked to stem cells and could be a contributing factor to healing capacity of stem cells [47, 48]. This potentially explains the preservation of the architectural structure of the parotid gland in the current study when exosomes were applied. The reduced fibrosis around the excretory ducts evident in exosome-treated groups could be attributed to the mechanisms described above. Other studies suggested more complex mechanisms of action for exosomes derived from human-extracted deciduous teeth which increase the paracellular permeability of glandular epithelial cells through Akt/GSK-3*β*/Slug pathway-mediated ZO-1 expression [49], and DPSC-derived exosomes via the GPER-mediated cAMP/PKA/CREB signaling pathway [33].

The immunomodulatory effects of exosomes are not limited to regulating inflammatory responses but also extend to exploiting the apoptotic signaling pathways. In our study, there was an increase in *BCL-2* which an antiapoptotic marker with exosomes. This is confirmed by a previous study that demonstrated a reduction in the apoptotic rate in Sjogren's syndrome after application of MSC-derived exosomes [44]. Furthermore, the downregulation of caspase-3, which is responsible for mediating apoptosis in our study is consistent with repair of parotid gland injury in diabetic rats that was achieved using the same mechanism [50]. Further possible effects of exosomes on the immune system include the control of complex signaling pathways that transform macrophages into the antiinflammatory instead of the proinflammatory phenotype [51]. This helped with the alleviation of periodontitis in mice and could be responsible for the morphological changes observed in blood vessels in exosome-treated groups in the present study. The current study revealed similar results to another study that investigated the effects of human DPSC-derived conditioned medium on the main excretory ducts of submandibular salivary glands after release from ligation procedures performed for treatment of sialolithiasis [52]. It should be noted that the mechanism of introduction of exosomes is different between both studies. This opens the horizon for exploring different approaches to introduction of exosomes to the salivary gland tissues, including intraglandular transplantation [53], or the development of an extracellular matrix/scaffold [39]. As the techniques of isolation of stem cells vary between laboratories and research institutions, it can be difficult to compare the efficacy of different stem cell-derived therapies due to the heterogeneity of methods. It has been suggested to isolate pure MSCs from the target organ even if the number of cells is limited within the adult tissue, which is consistent with the FDA concept of homologous use, using the same source of MSCs as the target organ [54]. Based on the above, using exosomes derived from MSCs native to the parotid salivary gland could have resulted in similar or possibly better results.

The upregulation of TNF-*α* that was observed in the current study with 5-FU is in alignment with another study that demonstrated oral, intestinal mucositis as well as increased levels of TNF-*α* [54]. Furthermore, the same study showed decreased cell proliferation and increased levels of apoptosis which is similar to the results obtained from the present study [55]. It is worth noting that the study mentioned above relied on a rat model that was solely based on chemotherapy in the absence of other noxious stimuli such as radiation, chemical, or mechanical injuries, to induce oral lesions. This approach is similar to what we implemented in our study design. Exosomes have been used successfully as therapeutic agents in treatment of radiation injuries in different tissues [56]. Therefore, it would be worth investigating the healing capacity of exosomes against a combined chemo-and-radiotherapy protocol simulating a real clinical scenario.

The results obtained from this study are also consistent with other studies in the field of medicine. For example, mesenchymal SC-Exo in cardiovascular and cerebrovascular diseases due to its superior properties in stimulation of blood vessels formation, stability, low toxicity as well as biological and immunological compatibility with human tissues [57, 58]. A recent study described the intranasal administration of SC-Exo for management of central nervous system diseases [59]. It was also shown that SC-Exo have therapeutic effects on autoimmune and neurodegenerative disorders [60], as well as orthopedics [61], diabetes therapy [62], and prevention of skin photoaging [63]. Other examples from the literature are the usage of exosomes in wound healing and management of skin injuries [64], orofacial tissue regeneration [65], as well as production of antimicrobial agents, management of microbial infections and pathogenic microbes [40, 66]. Recent reviews listed clinical applications of exosomes within 14 different disciplines of health [13], and moreover, the diagnosis, progression, and management of oral diseases [35]. Furthermore, MSC-derived exosomes have been described as an “antimicrobial weapon” against orodental infections [67]. They are gaining an increased popularity in nanomedicine as a powerful innovative tool for drug delivery [68]. In addition to the above, pluripotent stem cell-based therapies for salivary gland hypofunction have shown great success, which is highly relevant to this study [69]. Given the fact that the oral cavity is a very rich source of stem cells, regenerative dentistry applications seem plausible in the near future especially in restoring the periodontium and regenerating the dental pulp [70].

Future applications of exosomes include that they are considered as a safer replacement option for stem cell therapy that has been linked with tumorigenicity [68] as well as being a more practical option [71]. This is facilitated by a number of inherent properties of exosomes that allow them to target specific type(s) of tissues, or to be combined with specific ingredients serving as carrier molecules [72]. Furthermore, exosomes have superior properties to nanoparticles due to it higher stability, biocompatibility, and large-scale intercellular communications [68]. On the other hand, the diversity of exosomes and EVs remains as an obstacle to clinical translation, therefore, it was suggested that specific metrics need to be developed to characterize the different vesicles [73]. With special reference to salivary gland tissues that are relevant to the present study, exosomes have been proposed as a cell-free therapy to mitigate the injuries that resulted from irradiation of salivary glands [74]. Well-known materials used in clinical practice, such as hydrogel, are a result of combining exosomes with biomaterials [75], due to exosomes' unique ability to bind matrix proteins such as collagen type-1 and fibronectin [76]. The combination of exosomes with biomaterials opens the horizons for endless opportunities in the form of gradual/delayed release of exosomes over time [77] and supporting exosomes in a three-dimensional scaffold that offers longevity and mechanical support [78, 79].

5-FU was chosen in the current study as it is widely used as a chemotherapeutic agent and produced the most toxic effects on submandibular salivary glands of rats when compared to other chemotherapeutic agents, including cisplatin, methotrexate (MTX), and adriamycin (ADR) [80]. 5-FU remained detectable in saliva for up to 48 h after the completion of treatment [81] and is well known for negatively affecting salivary glands through inflammation, OS, alteration of the salivary composition, and reduction of the salivary flow [5]. For example, it has been demonstrated that 5-FU resulted in deterioration of the histopathological and immunohistochemical pictures of the lingual mucosa and submandibular salivary glands in rats [82]. Another study showed that 5-FU affects carbohydrates and its metabolism in the body, decreasing the use of glycogen as a substrate and a source of energy in the submandibular salivary glands of rats [83]. The above finding has been confirmed in the current study through histological and immunohistochemical examination as well as RT-PCR for evaluation of gene expression. Therefore, it is not surprising that 5-FU combined with other drugs failed to produce remarkable results in the treatment of advanced salivary gland cancer due to its cytotoxic effect on the glandular tissue [84]. As a result of the negative systemic effects of 5-FU, new approaches are being developed in an attempt to reduce the side effects of the chemotherapeutic medication including the recent development of 5-FU dissolvable layered microneedle patches for local application into areas of oral carcinoma [85]. This would result in higher local concentrations of 5-FU and potentially less systemic side effects.

Other potential methods for counteracting the cytotoxic effect of 5-FU on salivary gland tissues include Febuxostat, which is a Xanthine Oxidase Inhibitor, that works in a similar way to exosomes but also manipulates specific signaling pathways to achieve optimum functions [86]. Furthermore, electroacupuncture, which attenuated *TNF-α* expression was proposed as a protective and preventative measure against salivary gland damage caused by treatment with 5-FU [87]. Also, melatonin has been trialed as a protective agent to attenuate the effects of 5-FU on the lingual mucosa [88]. The dried root of *Salvia miltiorrhiza* Bunge (SM) (Lamiaceae), was helpful in protection against 5-FU-induced oral mucositis [89]. In addition to the above, amino acids [90], laser phototherapy [91], and an elemental diet [92] were used to protect against the changes induced by 5-FU in salivary glands, and dexamethasone-loaded PLGA nanoparticles reduced clinical signs and symptoms of oral mucositis induced by 5-FU [93]. These alternative methods could overcome the current obstacles that slow the development of standardization of isolation and biobanking procedures for exosomes [94] and its approval of investigational new drugs for clinical translation [95]. It is of extreme importance to note that there is a fine line between regenerative medicine and carcinogenic pathways. Therefore, since prospective patients that could benefit from exosome's therapeutic effects as the ones described in the current study are expected to have a history of cancer, the risks of cancer recurrence should be weighed against the anticipated anticytotoxic effects [54]. Breaking the code of the complex epithelial–mesenchymal interaction during the development of salivary glands is the key for loading miRNA signals into exosomes to coordinate formation and/or restoration of salivary gland tissue [96]. However, up to date, this remains a hurdle requiring extensive future research and investigations.

Exosomes have been proposed as antitumor growth agents that could have the potential to suppress tumors, however, this is still controversial [97]. Some studies showed that exosomes inhibit tumor cell production and increase the apoptotic cell rate [98–100], while other studies demonstrated that exosomes facilitate tumor growth [101, 102]. It has been demonstrated that exosomes could be helpful in delaying tumorigenesis in early oral premalignant lesions [103]. Also, exosomes derived from umbilical cord MSCs suppressed oral squamous cell carcinoma In vitro [104], while mesenchymal SC-Exo reduced the invasiveness of head and neck squamous cell carcinoma [105]. In the present study, *BCL-2* gene expression has increased significantly in the exosome group which is a double edge sword as besides the antiapoptotic properties which helped with the healing of the salivary gland, the increased *BCL-2* levels have also been linked with different types of cancer [106, 107]. Furthermore, the significant increase in *TGF-β* levels observed in the current study restored damage in the absence of a tumor in our animal model. However, downregulation of *TGF-β* has been linked with tumorigenesis in certain salivary gland cancer cell lines as well as being sensitive to certain anticancer drugs, including 5-FU and increasing its induced apoptotic properties [108]. In addition to the above, exosomes have demonstrated “tumor tropism,” which enhances the targeting of cancer cells and reduces the damage to normal cells [109]. For example, it has been shown that exosomes penetrate easily and integrate into cancerous cells, and in presence of prodrug 5-fluorocytosine, cause apoptosis in tumor cells [110]. Based on the above, it is hypothesized that exosomes could be used for targeted therapy of diffuse intrinsic pontine gliomas (DIPGs), which are fatal tumors affecting young children and are nonresponders to standard protocols of radiotherapy and chemotherapy [71]. Therefore, the results from this study as well as similar studies should be interpreted with caution as the exosomes' benefits of healing and repair should be weighed against the risks of cancer development.

The novelty of the current study is that it is the first to specifically investigate the therapeutic effects of bone marrow SC-Exo on 5-FU-induced cytotoxicity in the parotid gland, using a comprehensive approach involving histological, immunohistochemical, and molecular evaluation methods. This research fills a critical gap in understanding how to mitigate chemotherapy's adverse systemic effects on noncancerous tissues and has the potential to develop novel therapeutic strategies using bone marrow SC-Exo to protect against 5-FU-induced cytotoxicity in the parotid gland, thereby improving patients' quality of life during chemotherapy. Furthermore, the decreased expression of Ki-67 (a well-known prognostic marker for certain types of cancer) [111] after treatment with BMMSC-derived exosomes warrants further exploration of this novel approach as a means of slowing or stopping the excessive cell division and possibly opens the horizon for further experiments investigating the therapeutic potential for BMMSC-derived exosomes in treatment of salivary gland tumors. In addition to the above, the results obtained from our study regarding the downregulation of *TNF-α* and *iNOS* expressions suggest a potential therapeutic role for BMMSC-derived exosomes. This is supported by the Inhibition of gene expression and production of *iNOS* and *TNF-α* in neurodegenerative disorders stimulated by microglia [112].

In terms of our study design, saline was administered via oral gavage in Group I as injection-based administration (used in the experimental groups) carries its own stress-related physiological responses, including localized tissue reactions, which could introduce additional variables unrelated to chemotherapy-induced toxicity. Our intent was to establish a baseline condition in which the animals did not experience injection-related physiological stress, ensuring that any observed differences in the experimental groups could be attributed to the treatment rather than the mode of administration.

A limitation of the present study was that cancer was not induced in the rats before applying the different treatments (5-FU and BMMSC-derived exosomes) to simulate an authentic clinical scenario. The physiological body functions of the body could be significantly altered while dealing with malignancy. On the contrary, combined 5-FU and exosome therapy would potentially have a superior suppressive effect on the tumor progression and could achieve similar or even better results than the current study due to the possible higher uptake of 5-FU by the tumor cells. Another limitation is the lack of investigation of antioxidant markers expression, which could have contributed either directly or indirectly to the improvements observed within the 5-FU-treated parotid glands after treatment with MSSMC-derived exosomes. Furthermore, future research is deemed necessary to compare the effects of exosomes derived from dental pulp stem cells (DPSCs and MSCs native to the parotid salivary glands versus those derived from BMMSCs on parotid glands treated with 5-FU.

## 5. Conclusions and Future Research Recommendations:

Within the limitations of the present study, we conclude that BMMSC-derived exosomes can counteract the cytotoxic effects of 5-FU on the parotid glands of rats in vivo. Further research is warranted to confirm the exact mechanisms of action of BMMSC-derived exosomes as well as further explore the potential diagnostic, prognostic, and therapeutic application of BMMSC-derived exosomes in salivary gland neoplasms. Clinical trials or animal experiments after the induction of different types of tumors and simulating different combinations of radiotherapy and chemotherapy are required to confirm the findings from the current study and establish the possibility of clinical translation. Finally, utilizing stem cells that are native to the oral cavity and salivary glands as well as expression of other salivary glands tumors related markers such as Calponin, p63, CK14, Epithelial membrane antigen (EMA), and carcinoembryonic antigen (CEA) should be explored in future research.

## Figures and Tables

**Table 1:** Primer sequences used for PCR analysis.

Gene	Forward sequence	Reverse sequence
*TGFβ1*	CTACTATGCTAAAGAGGTCACC	TTTCTCATAGATGGCGTTGTTGC
*TNF-α*	AGCACAGAAAGCATGATCCGAG	CCTGGTATGAAGTGGCAAATCG
*BCL-2*	CACAAACCCCAAGTCCTCCTT	TGGAAGCCATTGCACTGAGA
*β-actin*	TCGTGCGTGACATTAAAGAG	ATTGCCGATAGTGATGACCT

## Data Availability

The data that support the findings of this study are available from the corresponding author upon reasonable request.
